# Performance of Sustainable Mortars Made with Filler from Different Construction By-Products

**DOI:** 10.3390/ma15072636

**Published:** 2022-04-03

**Authors:** Antonio López-Uceda, Enrique Fernández-Ledesma, José Ramón Jiménez, José María Fernández-Rodríguez

**Affiliations:** 1 Área de Ciencia de los Materiales e Ingeniería Metalúrgica, Leonardo Da Vinci-Campus de Rabanales, Universidad de Córdoba-Ed, 14071 Córdoba, Spain; p62louca@uco.es; 2 Área de Ingeniería de la Construcción, Leonardo Da Vinci-Campus de Rabanales, Universidad de Córdoba-Ed, 14071 Córdoba, Spain; efledesma@uco.es; 3 Área de Química Inorgánica, Universidad de Córdoba-Avda, de la Universidad s/n, 14240 Córdoba, Spain

**Keywords:** sustainable mortars, construction by-products, granite filler, ceramic filler, siliceous filler, recovery filler

## Abstract

One way to contribute to sustainability in the construction sector is through the incorporation of construction by-products from their own activities. This work intends to extend the possibilities for enhancement of these by-products through the incorporation of four different ones, as fillers, in mortar production. The influence of these incorporations in mortar production was compared with a reference mortar with siliceous filler in its fresh state; workability, entrained air content and fresh density, and in its hardened state; capillary water absorption, water vapour permeability and shrinkage (up to 91 days); and adhesive, compressive, and flexural strength; the last two were studied over time (up to 180 days). Despite the reduction in compressive strength, both in the short and long term, there was a gain in adhesive strength when the construction by-products were incorporated. Regarding the physical properties and durability studied, no relevant differences were found with respect to the reference mortar. According to the European Specifications, these mortars could be used as regular or coloured rendering and plastering mortars, and masonry mortars, and these findings promote the circular economy in the construction sector.

## 1. Introduction

The circular economy is based on integrating the use of waste as a secondary raw material, making it possible to face the environmental demands established by European policies. It has been indicated that the objective is to optimise natural non-renewable resources and waste recovery [1] in all economic areas.

The construction sector is well-known as a generator of waste related to unsustainable practices. However, integration of the use of secondary raw material generated from industrial activity faces environmental demands by state policies. In the European Union, an action plan has been set up for the circular economy, with the aim of including waste-as-a-resource business models [2].

In the construction sector, mortar is very important since it can have multiple uses, including repairing or binding building blocks, along with wall and ceiling coatings, which serve as a regularisation layer, to later be painted. Mortar is also used as glue to fix plates and coat walls or floors. Most multiple-use mortars are composed of cement, sand, and water in varying amounts, according to the area of use and application. Additionally, filler can be used for the purpose of acting as a site of nucleation and growth for hydrates and induce an acceleration of the hydration reactions due to the additional solid surface brought about by its powdery size particles [3].

The increasing generation of industrial wastes implies an issue to tackle from a sustainable point of view since its proper management is not occurring worldwide. The use of alternative materials instead of raw materials, such as sand, in mortar production reduces the consumption of natural aggregates and the amount of waste that remains untreated [4]. A vast number of by-products from the construction industry has been studied as sustainable materials in mortars. It has been reported that the use of sanitary ware of up to 20% of ratio replacement as filler in mortar production increases flexural and compressive strengths [5,6], and reduces shrinkage [5,7], compared to the reference mortar. In the research carried out by Esquinas et al. [8,9], the incorporation of filler from the drying process of aggregates in hot -mix asphalt plants—called Recovery Filler (RF)—in self compacting concrete presented lower mechanical properties and shrinkage than the mix with silicious filler. Concerning replacing sand by ornamental rock in mortars production, in Amaral’s et al. [10] study compressive strength in mortars increased as the incorporation ratio rose. This was attributed to the better particle size distribution of the aggregate, and Azevedo et al. [11] did not find any significant difference with up to 30% of this waste ratio incorporation, in general terms, in physical and mechanical properties. Mármol et al. [12] concluded that up to 10% of granite filler can be substituted without loss of compressive strength at 28 days, whereas Gupta and Vyas [13] obtained an increase in drying shrinkage and an increase in the adhesive strength when natural sand was replaced by powder granite. Other authors such as Torres-Gómez et al. [14] studied other wastes such as non-conforming fly ash from a coal-fired power plant as filler in mortars and recycled sand from masonry waste.

The use of waste, as well as of industrial by-products, in a cement-based materials such as mortar has been the subject of an extensive study. In most of the works related to the utilisation of solid wastes as raw material for mortar production, no more than two wastes were studied [4,15,16]. This work presents a study that deals with the incorporation of four different construction by-products in the production of sustainable mortars. For this purpose, a siliceous filler has been used as a reference mortar mix, which was replaced in the rest of the mixes by filler with different origins; ceramic from construction and demolition waste (CDW) treatment plants; waste from the drying process of the aggregate used in the manufacture of hot-mix asphalt; and granite and ornamental rocks, from their corresponding industrial processes (splitting, sawing, and polishing).

The overall construction and demolition waste generation was more than 3.0 billion tonnes per year until 2012 around the world [17]. CDW consists of ceramic particles, mortar, concrete, and natural aggregates, as well as minor amounts of asphaltic material; gypsum; and impurities such as wood, metal particles, paper, and plastics. In Spain, the generation of CDW was estimated to account for 14 Mt in 2016 [18], and ceramics represents, by far, the greatest proportion in comparison with other countries [19]. Ornamental rocks are widely used in buildings from all over the world for different purposes such as coat countertops, floors, or walls. It is estimated that Brazil produces around 8.2 Mt per year, generating 2 Mt to 2.5 Mt of waste, and represents 5–6% of world production [20]. In 2015, the Spanish production of ornamental rocks was around 3.5 Mt, representing 19% of world granite production [21]. In 2017, the European production of hot and warm mix asphalt was near 300 Mt, whereas in Spain it was 15.2 Mt; it is estimated that RF represents 4%, by weight, of world production [9,22].

The main objective of this work is to compare the effects of the use of different fillers from different construction by-products in the manufacture of mortars. These were compared to the reference through properties in a fresh state such as workability, entrained air content, and fresh density, and in a hardened state, such as capillary water absorption; water vapour permeability and shrinkage (up to 91 days) in terms of durability; and adhesive, compressive, and flexural strength; the last two were studied over a period of time (up to 180 days).

## 2. Materials and Methods

### 2.1. Characterisation of the Fillers Used

For this study, a siliceous filler (SF) was used in the reference mortar mixture. This filler was replaced, by weight, in the rest of the mixtures by recycled filler from different origins; ceramic from a nearby CDW treatment plant after being crushed in a Los Angeles test device after 3000 cycles with 11 steel balls of 425g ± 20 g weight per each one, Ceramic Filler (CF); waste from the drying process of the aggregate used in the manufacture of hot-mix asphalt, Recovery Filler (RF); sludge from a granite industrial process plant of splitting, cutting, and polishing, Granite Filler (GF); and ornamental rock waste from an ornamental rock sawing, Mixed Filler (MF); both sludges were dried in laboratory conditions before their use. All of them were collected from different locations close to the laboratory where this research was carried out. Table 1 shows the properties studied in laboratory of the fillers used. The Standard UNE-EN 13139:2003 establishes that the percentage passing through the 2 mm sieve must be 100%, through the 0.125 mm sieve over 85% and above 70% through the 0.063 mm sieve for fillers. According to this, GF and RF did not meet that requirement whereas the other fillers did. The chloride and sulphate content limits imposed by the EHE-08 were met by the fillers except the FC, whose sulphate content was above the limit. This was attributed to the gypsum detected in DRX test, as will be seen later. This is in accordance with authors such as Ledesma et al. [23] and Rodrigues et al. [24], who studied fine recycled sands from CDW with similar characteristics.

Table 2 shows the mineralogical phases of the fillers used, which were analysed by X-ray diffraction (XRD) using a Bruker D8 Discover A 25 with Cu Kα radiation (λ = 1.54050 A; tube voltage: 40 kV; Tube current 30 mA), and goniometric scanning was used from 10° to 70° (2θ°) at a speed of 0.05°/min. The main crystalline phase was quartz for the fillers studied except for the RF, in which dolomite was the only phase presented. The SF and RF presented silica and dolomite, respectively, as the only phases available. Otherwise, albite, calcite, and gypsum phases were detected in CF; albite, biotite, and microcline in GF; and biotite and cristobalite, to a lesser extent, in MF.

### 2.2. Heavy Metal Leaching Evaluation of the Fillers Used

A compliance test was carried out, according to standard UNE-EN-12457-4:2003, in order to determine the concentration of heavy metals in the leaching. The fillers used were classified according to the criteria established by EU Council Decision 2003/33/EC.

For conducting the standard UNE-EN-12457-4:2003, dry samples of each filler were taken of 0.090 kg. The content of 900 mL of deionised water was added into a PET container, along with the dry sample, to establish a liquid/solid ratio (L/S) equal to 10 l/kg. The samples were shaken in a tumbler for 24 h and, after, collected after passing through 0.45 μm filters. Table 3 shows the heavy metal elements leached conducting this test on the fillers used. Due to the high levels of sulphate content found previously (Table 2), sulphate anion was also studied for the CF, resulting in a concentration of 9590 mg/kg. The release levels of pollutant elements with the limits established by the Landfill Directive DC 2003/33/EC enable the classification of materials according to their hazardous potential. Due to the release levels of Mo in GF, Sb and Hg in MF, and sulphate anion in CF, these fillers were classified as non-hazardous materials, whereas SF and RF were classified as inert waste. These non-hazardous classifications are in agreement with other research [23,25].

### 2.3. Experimental Programme and Methods

Mixtures, named as in Table 1, were produced in the laboratory. The dosage for the manufacture of the mortar mixtures was made in proportion by weight: 3500 g of natural sand, 500 g of cement, and 300 g of filler. The amount of water was adjusted experimentally to achieve a consistency of 175 ± 10 mm (UNE-EN 1015-3:2000), which is a suitable workability for masonry and rendering applications (UNE-EN 1015-2:1999). A 0.1 mL of commercial plasticiser (NEOPLAST) was added to the water. The water to cement plus filler ratio was kept constant between 0.85 and 0.90. The cement used was CEM I 52.5 R. The mixing process was defined in previous research by Jiménez et al. [26]. A standard mixer was used according to UNE-EN 196-1:1996.

Table 4 summarises all the tests conducted; four specimens were used for each test. The evolutions of mechanical characteristics, and compressive and flexural strength, were studied over time after 7, 28, 91, and 180 days of curing. Also studied was the adhesive strength and density in hardened mortar at 28 days of curing. Shrinkage was measured after 1, 7, 14, 28, 49, and 91 days of curing. Samples were stored before being tested in a chamber at constant temperature (20 °C ± 2 °C) and relative humidity of 65 ± 5% after 24 h of manufacturing.

## 3. Results and Discussion

### 3.1. Properties in Fresh State

**Figure 1:**
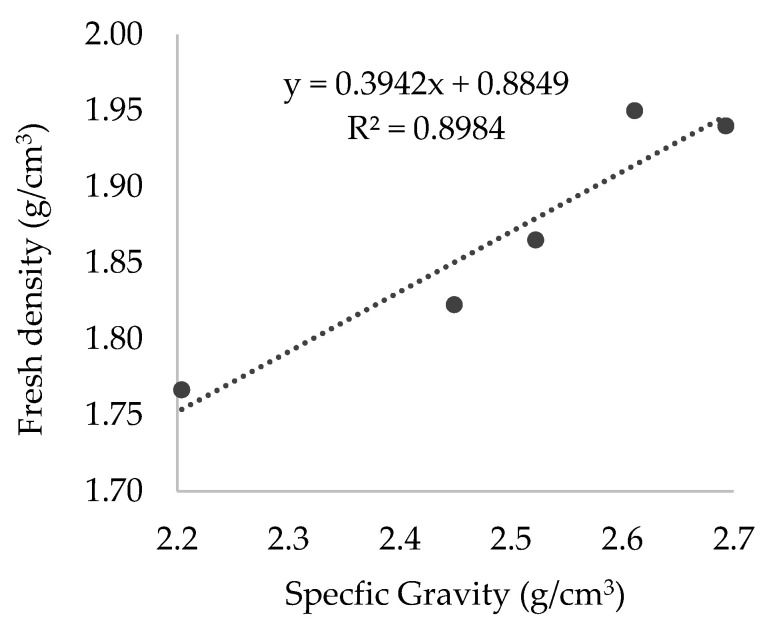
Correlation between fresh density in mortars and specific gravity of the fillers used in mortars.

Table 5 shows the properties studied of the fresh state such as workable life, density, and entrained air content in the mortars produced. Workable life determines the period of time when a mortar has a sufficient and an appropriate consistency to be used without any additional water, while the mortar must not harden for its correct use. The replacement of SF by the fillers used shows that the workable life decreases. The percentage of reduction compared to M-SF ranged from less than 7% in the case of M-RF to more than a half in M-MF. Studies on the incorporation of recycled CD&W found a decrease in this test [23,26]. The standard followed does not establish any range for its acceptance [27]. The entrained air content test consists of pouring water on the surface of a known volume of mortar and applying an air pressure that forces water into the mortar, displacing the air within the pores. Test results were alike, except the M-MF, which presented the lowest value (2.7%). Similar values were found by Zhao et al. [28], studying the incorporation of ceramic filler in self-compacting mortars. Amaral et al. [10] also found a decrease in air content when incorporating ornamental stone waste, attributed to the strong difference in density of substituted materials, as occurred in this research. The fresh density values in mortars are strongly related to the density of the filler used (Figure 1), as expected. Specifications for mortars for masonry EN 998-1:2018, and 998-2:2018 for rendering and plastering, do not establish any threshold for these tests.

### 3.2. Properties in Hardened State

#### 3.2.1. Physical Properties and Their Effects on Durability

Table 6 shows the properties studied related to durability, such as capillary water absorption and water vapour permeability. The capillary water absorption represents the pores that are interconnected with the external environment, forming large internal capillaries within the matrix, where water transportation might occur, along with gases. The capillary water absorption increased by 23% in M-RF, whereas it was reduced by 22% in M-FM compared to the reference mortar; no strong affection was found for the other mixes. The highest value presented by M-RF may be caused by the fact that RF´s particle size distribution was the greatest, so the less compacted cementitious matrix was formed, allowing for the raising of water by capillaries. This property is directly related to durability, making the use of mortars unfeasible when great values are obtained [11]. It has been reported that greater capillary water absorption values than 3.5 Kg/(m^2^/min^0.5^) impair the performance of mortars at medium and long term [29]; all values presented were lower than that threshold. EN 998-1 Specification sets a maximum of 0.2 or 0.4 Kg/(m^2^·min^0.5^) for class W_c_1 and W_c_2, respectively, according to the capillarity water absorption classification, for its use as coating mortar, repair mortar, or thermal isolation mortar, and no restriction for regular, light, or coloured rendering and plastering mortar. The permeability to water vapour is a feature that provides information about how it allows drying of the water within the render and reduces the risk of condensations within the wall and in the interior space [30]. The water vapour permeability was reduced in all mortars compared to the reference, with 35% being the greatest decrease in M-MF. It has been reported that the incorporation of fine wastes, such the ones that come from C&DW [6,31,32] or fly ashes from peat and wood [33], was responsible for the decrease in water vapour permeability. EN 998-1 establishes a minimum of 1.3·10^−11^ kg/m·s·Pa for being considered as repair mortar and thermal isolation mortar. Capillarity water absorption and water permeability test results are not limited to the use of mortars in internal walls or bonding bricks [34]. The results of hardened density did not vary significantly (below 3%) with respect to the reference mix, except for the mortar with CF incorporation, whose value was nearly 5% lower.

**Figure 2:**
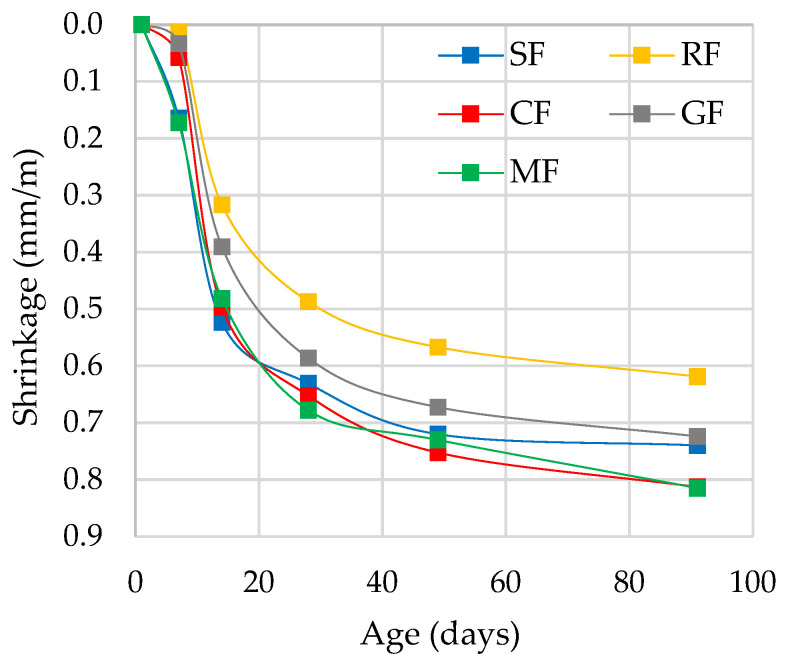
Shrinkage of mortars over time.

Shrinkage is a phenomenon that causes volumetric variation in cementitious base materials, which makes it one of the most significant aspects of mortar durability. Shrinkage values represent the combination between the self-desiccation of the mix during hydration and the loss of evaporable water in the mix to the environment. Figure 2 shows the shrinkage results for the five mixes after 91 days. Zhang et al. [35] studied the affection of aggregates on drying shrinkage in mortars, reporting that 91 days drying time can be used to analyse the influence of aggregates. The obtained shrinkage performance was consistent with the findings of several other researchers. In Torres-Gómez´s et al. [14] study about the incorporation of CF in mortars, shrinkage rise after 90 days was negligible for the mortars produced. The mean of the shrinkages at 28 days relative to 91 days of the mortars studied was 81.7 ± 3.6% (Figure 2). It is shown that the shrinkage in M-MF and M-CF was 10% higher than in M-SF (ref mix), while it was reduced by 16% in M-RF. This means that low differences were presented among the shrinkage curve mortars. Itim et al. [36] found that the presence of different mineral additions in mortars, such as limestone powder and natural pozzolan, did not substantially affect the drying shrinkage at long-term. The M-FR presented the lowest shrinkage at 91 days, which may be due to the coarser particle size presented by RF. This in accordance with Esquinas et al. [9] and Jackiewicz-Rek et al. [5]. None of the mortars exhibited values above the typical ones presented by OPC mortars (1 mm/m) [37]. Due to all of that, it has been proven that the shrinkage behaviours of the mortars produced were adequate.

#### 3.2.2. Mechanical Properties

The adhesive strength performances were alike for all the mixes; M-CF, M-GF, M-MF, and M-RF test results/standard deviations were 0.33/0.04, 0.31/0.06, 0.38/0.10, 0.29/0.05 MPa respectively, expect for M-SF, which presented a lower value, 0.18/0.08 MPa. It has been reported that the finer particle size distribution in aggregates reduced the adhesive strength in mortars [38]; this supports the results since siliceous filler presented the finest size. The EN 998-2:2018 specification for mortars establishes a minimum value of 0.3 MPa if the mortar is used as rendering or plastering, and 0.15 MPa as a masonry mortar, EN 998-1:2018. All mixtures complied with the limit for the masonry mortar. The fact that the adhesive strength test results were higher in mortars with filler replacement was in accordance with the ones presented by other authors. Silva et al. [39] and Jiménez et al. [26] reported that the effect of the incorporation of similar material in CF was caused by the pozzolanic effect of the ceramic fines. Kabeer et al. [40] also found an increment in mortars incorporating marble characterised by dolomite as the only mineralogical phase detected, as in RF (Table 2), along with Gupta and Vyas [13], who studied natural aggregate replacement by granite powder in mortars.

**Figure 3:**
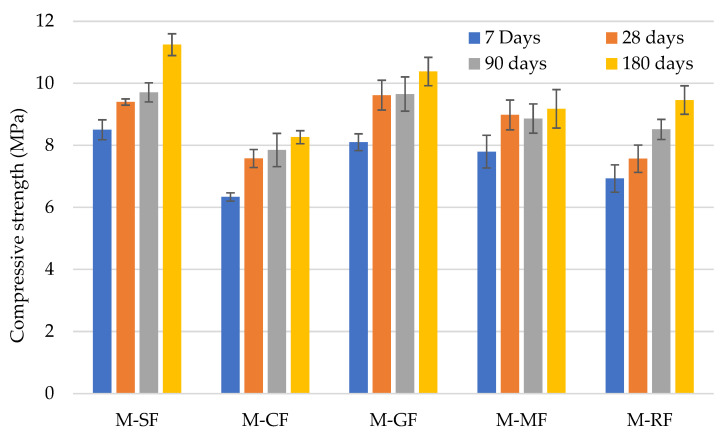
Compressive strength of mortars over time.

Regarding the compressive strength, all compressive strength test results of the mortar mixtures were above 7.5 MPa after 28 and 90 days, and 8 MPa after 180 days (Figure 3). The fact that the compressive strength at 28 days was greater than 7.5 MPa for all the mixes implies that they could be classified commercially as M7.5 [41]. Regarding the EN 998-2:2018 specification, all mortars would be classified as M5 (>5 MPa), while according to EN 998-1:2018, mortars would be classified as CS IV (≥6 MPa). The test result values were lower, in general, in mortars with filler replacement than the ones of reference mortar at the same age. This can be explained by the fact that, on the one hand, the intensity of pozzolanic reaction that happened in reference mixture with SF [8] did not occur in the rest of mixes due to the SF greater relative presence of SiO_2_ and, on the other hand, by the filler effect due to the lower particle size of the SF.

Nasr et al. [42] studied cement mortars with partial substitution of cement by different wastes such as marble, granite, porcelain tiles and clay bricks, finding a negative impact on their mechanical properties compared to the reference mix. In our study, the greatest reduction occurred in M-FC with 26.5% at 180 days, whereas marginal differences in M-GF were achieved with respect to the control mortar at the different ages studied; in agreement with the findings of Ramos et al. [43] and Ledesma et al. [23] respectively. Regarding M-GF, the presence of pozzolanic reaction in mortars with GF has previously been reported [44,45], which agrees with the results since M-GF performance was similar to ref mix. In mortars made with ornamental rocks, pozzolanic activity was not found as in the reference mortar [46]. Despite the founding of the pozzolanic reaction when ceramic filler was incorporated [47], authors such as Zhao et al. [28] obtained a decrease when incorporating ceramic filler in mortars, with respect to reference mix. Regardless of the absence of SiO_2_ in RF, it has been reported that dolomite can reduce the nucleation barrier, enhancing the hydration process [40]. Hence, somehow, the incorporation of the fillers used can help the formation of nucleation sites; whether pozzolanic reactions occurred or not, since the mechanical properties were not significantly affected.

**Figure 4:**
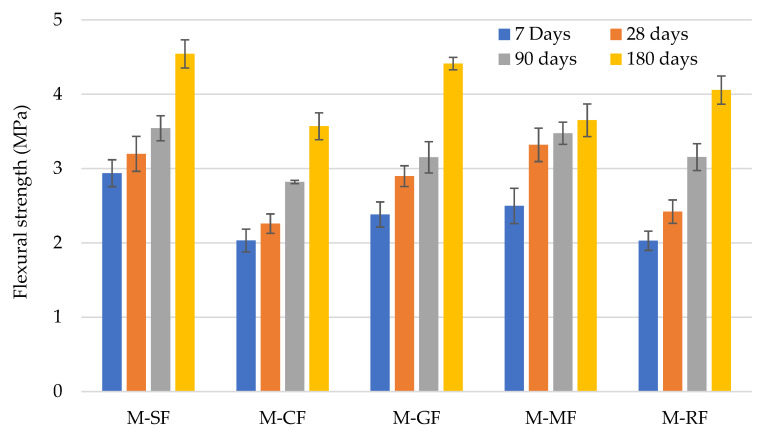
Flexural strength of mortars over time.

The performance of flexural strength (Figure 4) was analogous to compressive strength, which leads to the idea that no relevant difference in influence of the construction by-products’ incorporation in mortars from the compressive strength was exhibited.

**Figure 5:**
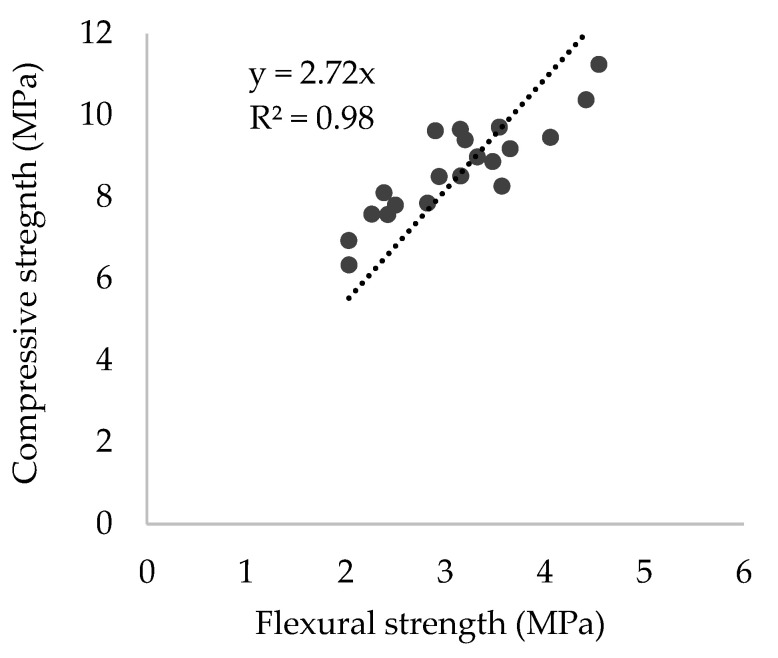
Correlation between flexural strength and compressive strength.

As part of linear regression between the flexural x axis and the compressive y axis, strength values were assessed (intercept or constant value equals 0), with a strong correlation found (R^2^ = 0.98) (Figure 5). It can be observed that the mortar mixes containing different construction by-products as fillers behave in a similar manner to that of the reference mortar. Compressive strength of all mixes resulted in 2.72 times the flexural strength; this agrees with Segura et al. [48], who found an almost identical relationship studying mortars with different limestone filler content.

## 4. Conclusions

The incorporation of the different construction by-products studied as filler in mortar has been studied as a viable use. Considering the results and discussion, the main conclusions reached are presented as follows:

- The fresh properties presented by the mortars produced were not substantially affected by the incorporation of the different construction by-products used as fillers compared to the reference, except the mortar with GF, which exhibited a relevant lower workable life and air entrained content. Despite the fact that the EN 998-1 and EN 998-2 specifications do not set any limit for these test methods, the addition of an additive agent in mortar mixes with ornamental rock sludge (M-MF), in order to enhance performance, could be interesting;
- In terms of the physical properties studied and their effects on durability, no relevant differences were found compared to the reference mortar. Capillarity water absorption differences in the mortars with respect to reference mix were ±23%, whereas in water vapour permeability, reference mix presented the highest value and M-MF a reduction of 35% with respect to it. The shrinkage of all the mortars studied was lower than 1 mm/m, which is the value usually presented by OPC mortars, and slight differences were found among them. Regarding the specifications EN 998-1 and EN 998-2, the use of the mortars produced would be restricted to regular, light, or coloured rendering and plastering mortar or bonding bricks and discarded as coating, repair, or thermal isolation mortars;
- With respect to the mechanical behaviour of the mortars produced, adhesive strength exhibited by the mortars with the different construction by-products as fillers was higher than the reference one, its use being valid as rendering plastering, according to EN 998-2, while the reference one was not. The incorporation of the construction by-products negatively affected the compressive and flexural strength. Despite this, all mortars would be commercially classified as M7.5, and, with respect to EN 998-1, Class CS IV would be proposed for regular, coloured, and coating rendering and plastering mortar. Over time, a similar performance was exhibited by all the mortars, achieving greater values of 8 MPa after 180 days.

Therefore, the use of construction by-products studied in mortar manufacturing could be a viable alternative to help increase sustainable development in the construction sector. Conforming to the specifications referenced and the properties studied, all mortars with construction by-product incorporation could be used as regular or coloured rendering and plastering mortars, and masonry mortars. The findings of this study can help one to reduce natural aggregate consumption, promoting new uses for construction by-products in the new circular economy paradigm.

## Figures and Tables

**Table 1:** Filler properties and mixture names.

Characterization Test Methods	Siliceous SF	Ceramic CF	Granite GF	Mixed MF	Recovery RF
Specific gravity (UNE 80103:2013) (Mg/m^3^)	2.61	2.52	2.45	2.20	2.69
Bulk density (1097-3:1999) (Mg/m^3^)	0.74	0.95	0.50	0.52	1.33
Chloride (UNE-EN 1744-1:2010) (%)	0.01	0.02	0.03	0.01	0.01
Acid Soluble sulphates (UNE-EN 1744-1:2010) (SO_3_%)	<0.01	0.98	<0.01	<0.01	<0.01
Total sulphurs (UNE-EN1744-1:2010) (SO_3_%)	<0.01	0.98	<0.01	<0.01	<0.01
Sieve size (mm)					
0.25 (% passing)	100	100	100	100	100
0.125 (% passing)	100	100	97.35	99.49	75.91
0.063 (% passing)	87.33	73.81	27.58	81.36	44.94
Mortar mixture names	M-SF (ref)	M-CF	M-GF	M-MF	M-RF

**Table 2:** Mineralogical analysis of fillers.

	Mineral Relative Abundance (*)				
Mineral Phases	SF	CF	GF	MF	RF
Albite Na(Si_3_Al)O_8_	-	**	***	-	-
Calcite CaCO_3_	-	**	-	*	-
Dolomite CaMg(CO_3_)_2_	-	-	-	-	******
Illite KAl_2_Si_3_AlO_20_(OH)_2_	-	-	-	-	-
Quartz (SiO_2_)	******	****	*****	*****	-
Sanidine (Na,K)(Si_3_Al)O_8_	-	***	-	-	-
Gypsum CaSO_4_·2H_2_O	-	*	-	-	-
Biotite K(Mg,Fe)_3_ AlSi_3_O_10_(OH,F)_2_	-	-	****	*	-
Microcline KAlSi_3_O_8_	-	-	**	-	-
Cristobalite SiO_2_	-	-	-	**	-

Footnote: The greater number of asterisks (*), the more relative abundance is found in the mineralogical phase; from *, almost negligible, to ******, the exclusive mineralogical phase observed.

**Table 3:** Leached concentrations of fillers (mg/kg) and acceptance criteria (WAC, EU Council Decision 2003/33/EC).

						Criteria EU LD 2003/33/EC (L/S = 10)		
Elements	SF	CF	GF	MF	RF	Inert	Non-Hazardous	Hazardous
Cr	n.d.	0.344	n.d.	0.006	n.d.	0.5	10	70
Ni	n.d.	0.026	n.d.	0.040	0.004	0.4	10	40
Cu	n.d.	0.039	n.d.	0.862	n.d.	2	50	100
Zn	n.d.	0.010	n.d.	0.016	0.011	4	50	200
As	0.018	0.030	0.014	0.043	0.003	0.5	2	25
Se	0.003	0.042	n.d.	0.034	n.d.	0.1	0.5	7
Mo	n.d.	0.088	0.565 ^(1)^	0.052	0.020	0.5	10	30
Cd	n.d.	n.d.	n.d.	n.d.	n.d.	0.04	1	5
Sb	n.d.	0.035	0.006	0.489 ^(1)^	0.024	0.06	0.7	5
Ba	3.746	0.498	0.010	0.833	0.272	20	100	300
Hg	n.d.	n.d.	n.d.	0.122 ^(1)^	n.d.	0.01	0.2	2
Pb	n.d.	n.d.	n.d.	0.000	n.d.	0.5	10	50
C (µS/cm)	28.75	785	129.8	118.2	40.4			
T^a^ (°C)	25.7	25.9	28.1	28.3	28.1			
pH	9.05	9.51	9.05	9.02	9.41			

^(1)^ Exceeds the inert waste limit; n.d. non detected.

**Table 4:** Tests performed to study the properties of the mortars produced.

Test Methods	Standards
Workable life	UNE-EN 1015-9:2000
Entrained air content	UNE-EN 1015-7:1999
Density in fresh state	UNE-EN 1015-7:1999
Density in hardened mortar	UNE-EN 1015-10:2000
Adhesive strength	UNE-EN 1015-12:2016
Compressive strength	UNE-EN 1015-11:2000
Flexural strength	UNE-EN 1015-11:2000
Capillary water absorption	UNE-EN 1015-18:2003
Water vapour permeability	UNE-EN 1015-19:1999
Shrinkage	UNE 83831:2010 EX

**Table 5:** Properties in fresh state of the mortar mixes produced.

Test Methods	M-SF	M-CF	M-GF	M-MF	M-RF
Workable life (min)	206.5 (2.5)	179.8 (15.6)	152.3 (24.0)	92.3 (6.9)	192.5 (5.5)
Fresh density (g/cm^3^)	1.95 (0.02)	1.87 (0.01)	1.82 (0.03)	1.77 (0.02)	1.94 (0.01)
Entrained air content (%)	7.80 (0.70)	6.80 (1.70)	5.20 (2.10)	2.70 (0.70)	8.00 (3.10)

Standard deviation in brackets.

**Table 6:** Some properties in hardened mortar mixes.

Test Methods	M-SF	M-CF	M-GF	M-MF	M-RF
Hardened density (g/cm^3^)	1.76 (0.02)	1.67 (0.01)	1.80 (0.01)	1.72 (0.01)	1.71 (0.02)
Capillary water absorption (Kg/(m^2^·min^0.5^))	1.01 (0.04)	1.03 (0.02)	1.08 (0.03)	0.79 (0.03)	1.24 (0.06)
Water vapour permeability (10^−11^ kg/m·s·Pa)	1.11 (0.11)	1.01 (0.17)	0.85 (0.04)	0.72 (0.03)	0.9 (0.10)

Standard deviation in brackets.

## Data Availability

Not applicable.
